# Levothyroxine overdose in a hypothyroid patient with adjustment disorder: A case report

**DOI:** 10.1016/j.amsu.2020.09.045

**Published:** 2020-10-09

**Authors:** K.C. Kiran Kumar, Nirmal Ghimire, Trishant Limbu, Robin Khapung

**Affiliations:** a Department of Anesthesiology and Critical Care Medicine, Nepal Police Hospital, Kathmandu, Nepal; b Department of Internal Medicine, Nepal Police Hospital, Kathmandu, Nepal; c Department of Critical Care Medicine, Grande International Hospital, Kathmandu, Nepal; d Department of Anesthesiology and Critical Care Medicine, Karnali Academy of Health Science, Jumla, Nepal

**Keywords:** Levothyroxine overdose, Levothyroxine poisoning, Propranolol, Psychiatric illness

## Abstract

**Introduction:**

Levothyroxine (T4) overdose is not frequently encountered and for the clinical signs to materialize, the ingested dose, the rate of conversion of T4 to T3 and chronicity of overdose can be held accountable.

**Case report:**

A 29-year-old female, a known case of hypothyroidism and adjustment disorder, under levothyroxine, propranolol and sertraline, intentionally ingested 2.5 mg of levothyroxine but remained asymptomatic with sudden surge in T4 in initial hours of ingestion which gradually started declining along with reciprocal change in TSH. However, the change in T3 was almost negligible.

**Discussion:**

T3, the active thyroid hormone, when in excess accounts for toxic effects. The duration during physiological process of deiodination and half life of hormone correlates with onset and duration of symptoms. Propranolol which blocks peripheral conversion of T4 to T3 and sertraline which is also reported to reduce the efficacy of levothyroxine, which is evident from low T3 in thyroid profile, must have led to patient being asymptomatic despite lack of early gastric decontamination.

**Conclusion:**

Levothyroxine overdose up to 4mg/day may be asymptomatic but in patients with concomitant neurotic or psychiatric illness, who intentionally take it, are also put on drugs like sertraline and propranolol, the effects of which on thyroid hormones must be contemplated for possible explanation of being asymptomatic.

## Highlights

• Thyronorm overdose or poisoning by accidental ingestion may be seen in pediatric age group but is very rare in adult population.
• Overdose due to suicidal ideation may be the cause but appearance of toxicity depends on ingested dose, disease chronicity and amount of active hormone.
• Concomitant psychiatric illness and drug interaction with thyroid hormones should always be considered.

## 1 Introduction

Levothyroxine (T4) overdose is not a common entity and it can occur accidently, mostly in children, and can happen intentionally in adults with suicidal ideation especially psychiatric patients [1].

The common clinical signs following thyroxine overdose can either be limited to tachycardia, agitation, nervousness, insomnia, anxiety, tremor [2], or severe features, though less likely, like thyroid storm involving cardiac, neurological, respiratory and thermoregulatory center [3]. Acute levothyroxine ingestion up to a dose of 4 mg/day is usually asymptomatic and well tolerated [2]. Symptoms of thyrotoxicosis are evident in chronic over dosage cases which is also associated with higher morbidity and mortality [4]. In an established case of thyrotoxic crisis, propranolol, glucocorticoids, thionamides, iodine compounds, bile acid sequestrants [5], and in some cases plasmapheresis is also opted [6] along with other supportive measures.

We report a case of asymptomatic levothyroxine over dosage (2.5mg) in a chronic hypothyroid patient who also was concomitantly taking propranolol and sertraline for adjustment disorder.

### 1.1 Case report

A 29 years old female, a known case of hypothyroidism for past 7 years and adjustment disorder for past 3 months presented to Emergency Department with history of intake of 2.5mg of levothyroxine (100 tabs of 25mcg) 6 hours back and loss of consciousness for 1 hour, nearly 4 hours back. She had a verbal argument with her husband following which she took that medication. She was initially taken to a nearby health center but was immediately referred to our center without any primary management. She was on levothyroxine 50 mcg for hypothyroidism and sertraline 50mg along with propranolol 20 mg for Adjustment disorder.

On arrival at the Emergency Department, she was confused, her GCS was 14/15 (E4M6V4). Vitals were stable with a pulse of 84/min, regular, BP- 120/90 mm of Hg, Respiratory rate of 20/min, the temperature of 98.2 °F and SpO2 of 98% in room air. She was shifted to ICU for monitoring. She complained of mild headache and tingling sensations in her body. Her vitals were stable during the ICU stay and there were no clinical features of hyperthyroidism. Arterial Blood Gas analysis showed pH: 7.34, pco2: 28 mmHg, HCO3: 20 mmol/L, pO2: 78 mmHg, Lactate:0.8 mmol/L. However, her lab showed increased T4 post-ingestion which subsequently decreased in the following days. She was consulted with a psychiatrist and clonazepam was added apart from her sertraline and propranolol. Her ICU stay was uneventful and was transferred to ward after 72 hours and discharged home after counseling, 2 days later (Table 1).

**Table 1:** Thyroid profile.

	12 hours after ingestion	36 hours after ingestion	60 hours after. ingestion	Reference range
FreeT3	3.7	3.6	3.5	1.2–4.1 pg/ml
FreeT4	26	19.7	15.3	8.9–17.1 pg/ml
TSH	4	0.5	1	0.3–4.5 μIU/L

## 2 Discussion

Accidental Levothyroxine poisoning is more common in the pediatric population [7] than adult age groups where the overdose is more likely suicidal considering the collateral psychiatric illness but the case reports are merely handful [2,8,9]. Also, hypothyroidism is found to be associated with mood disorder [10].

Levothyroxine T4 is ultimately converted to T3 which is a biologically active part of thyroid hormone and is responsible for any adverse effects when in excess. This process of deiodination usually takes 24–48 hours which explains why patients are asymptomatic during the initial presentation and may persistently remain symptomatic for over a week considering the half-life of the hormone [11]. Thus the expected biochemical profile is increasing total and free T3, T4, and decreasing TSH which probably will be normalized over a week. Acute ingestion of levothyroxine up to 4 mg is usually asymptomatic [2]. However, some studies claim no relationship between the severity of symptoms and dose [12]. But, it can pertain that at higher doses the adverse features are more likely to occur, as in a study of 6 cases after ingestion of 7–12 mg of levothyroxine, five were comatose and one was stuporous. Three cases with arrhythmia and two presented with left ventricular failure [13]. Though our patient had presented late to tertiary center and even without gastric decontamination she remained asymptomatic despite her T4 rose to 26 pg/ml because the ingested dose was only 2.5 mg and secondly there was concomitant use of 20 mg of propranolol which also might have blocked the peripheral conversion of T4 to T3 which is also evident from the values of T3 which remained almost static after 60 hours of ingestion and hence asymptomatic [11]. Also considering sertraline, it has been reported that sertraline reduces the efficacy of levothyroxine in patients treated for hypothyroidism [14]. A study demonstrated that though sertraline was not associated with clinically significant changes in thyroid function, a significant amount of reduction of T3 and T4 were noted after 15 and 30 days of treatment [15]. So, the effect of sertraline on thyroid hormones also cannot be overlooked in this case which might have contributed in a way for the patient to remain asymptomatic.

## 3 Conclusion

Psychiatric patients with hypothyroidism may intentionally consume levothyroxine tablets with suicidal notion. Besides, the dose of Levothyroxine ingested, the use of concomitant drugs like sertraline and propranolol should also be taken into account while considering the signs of toxicity as these drugs may counter effect and hence the thyroid profile and clinical picture may not resemble overdose.

## Learning points

Psychiatric illness often accompany drug overdose or poisoning. The counter effects of those concomitant drugs like propranolol and sertraline are always to be considered as a cause in a patient with thyronorm overdose for being asymptomatic.

## Provenance and peer review

Not commissioned, externally peer reviewed.

## Sources of funding

There is no any source of funding for this case report.

## Ethical approval

This study was conducted in accordance with ethical standard and informed written consent was taken from patient for publication of this case report.

## Consent

Written informed consent was obtained from the patient for publication of this case report and accompanying images. A copy of the written consent is available for review by the Editor-in-Chief of this journal on request.

## Author contribution

1 Kiran Kumar KC wrote the report. And he was directly involved in patient's care during his stay in ICU.
2 Nirmal Ghimire took history, performed examination, sent relevant investigations and revised it with relevant references. And he was directly involved in patient's care during his stay in ICU.
3 Trishant Limbu provided support and mentorship for development, writing and revision of this case report. He was not directly involved in patient's care.
4 Robin khapung worked for literature review and revision of the case report into its final version. He was not directly involved in the patient's care.

## Registration of research studies

Name of the registry:
Unique Identifying number or registration ID:
Hyperlink to your specific registration (must be publicly accessible and will be checked):

## Guarantor

Kiran Kumar K.C. He is the first author and corresponding author for this case report.

## Declaration of competing interest

There is no any conflicts of interest.
